# Discovery of Differentially Expressed MicroRNAs in Porcine Ovaries With Smaller and Larger Litter Size

**DOI:** 10.3389/fgene.2022.762124

**Published:** 2022-02-09

**Authors:** Gaoxiao Xu, Yamei Hu, Dongling Yu, Xingfa Chen, Xiao Li, Saixing Duan, Ning Zhang, Gaoyu Xu, Jianhong Hu, Gongshe Yang, Shiduo Sun, Yong Liu

**Affiliations:** ^1^ Key Laboratory of Embryo Development and Reproductive Regulation of Anhui Province, Fuyang Normal University, Fuyang, China; ^2^ Teaching and Research Section of Biotechnology, Nanning University, Nanning, China; ^3^ Shaanxi Key Laboratory of Molecular Biology for Agriculture, College of Animal Science and Technology, Northwest A and F University, Yangling, China; ^4^ Nanning Dabeinong Feed Technology Co., Ltd., Nanning, China

**Keywords:** microRNA, pig, ovary, litter size, RNA-seq

## Abstract

The number of live births in a litter is an important reproductive trait, and is one of the main indicators which reflect the production level and economic benefit of a pig farm. The ovary is an important reproductive organ of the sow, and it undergoes a series of biological processes during each estrous cycle. A complex transcriptional network containing coding and non-coding RNAs in the ovary closely regulates the reproductive capability of sows. However, the molecular regulation mechanisms affecting sow litter size are still unclear. We investigated the expression profiles of microRNAs (miRNAs) in porcine ovaries from sows with smaller than average litter sizes (SLS) and those with larger litter sizes (LLS). In total, 411 miRNAs were identified, and of these 17 were significantly down-regulated and 16 miRNAs were up-regulated when comparing sows with LLS and SLS, respectively. We further characterized the role of miR-183 which was one of the most up-regulated miRNAs. CCK-8, EdU incorporation and western blotting assays demonstrated that miR-183 promoted the proliferation of granulosa cells (GCs) in pig ovaries. Moreover, miR-183 inhibited the synthesis of estradiol in GCs and promoted the synthesis of progesterone. These results will help in gaining understanding of the role of miRNAs in regulating porcine litter size.

## Introduction

The number of live births in a litter is an important reproductive trait that reflects the production levels and economic benefits of pig farms. The ability to increase the number of offspring in litters has always been a global goal for pig producers, and greater litter sizes and shorter delivery intervals have increased the number of live births per sow per year. This is the main factor which affects the economic benefits of sow farming (Huang, et al., 2016; Ma, et al., 2018; Xu, et al., 2019; Bakoev, et al., 2020; Zheng, et al., 2020). The ovariesy are important reproductive organs of female animals, and the reproduction ability of sows is strictly regulated by a complex transcriptional network of coding and non-coding RNAs within these organs (Liang, et al., 2013; Soh, et al., 2015; Terenina, et al., 2017; Xu, et al., 2019).

MicroRNAs (miRNAs) are single-stranded small-molecule non-coding RNAs composed of about 21–23 nucleotide bases, which are processed by Dicer enzymes from single-stranded RNA precursors with a hairpin structure of about 70–90 bases in size (Starega-Roslan et al., 2015; Svobodova et al., 2016). Although miRNAs are different from siRNAs, but they are closely related. miRNAs are involved in regulating the expression of one-third of mammalian genes, but their mechanism of action is different from siRNA-mediated mRNA degradation (Li, et al., 2019; Chen, et al., 2021; Huang, et al., 2021). Recent studies have shown that miRNAs are involved in the regulation of mammalian reproduction, especially ovarian function. For example, the expression of miR-17-5p and let-7b are lost in Dicer1-deficient mice, thereby reducing angiogenesis in the corpus luteum and thus showing female infertility in these animals (Liu, et al., 2018). When mouse granulosa cells (GCs) were treated with human chorionic gonadotropin (hCG), the expression of miR-132 and miR-212 were upregulated, and knocking out these miRNAs increased the expression of C-terminal binding protein 1 (CtBP1) in these cells (Fiedler, et al., 2008). Previous studies have found that the ovaries of Yorkshire pigs with smaller and larger litter sizes (SLS and LLS) can express a large number of miRNAs, and a total of 37 differentially expressed miRNAs were obtained, of which 21 were upregulated and 16 were downregulated in LLS relative to SLS that could be potentially used to increase porcine ovulation rate and litter size (Huang, et al., 2016; Ma, et al., 2018; Xu, et al., 2019; Bakoev, et al., 2020; Zheng, et al., 2020). Furthermore, it has been reported that there are several miRNAs, such as miR-21, miR-1306, miR-181a, miR-92a and miR-26b, that could affect sow fertility progression by regulating porcine GCs states (Liu et al., 2014a; Liu et al., 2014b; Choi, et al., 2016; Pan and Li, 2018; Yang, et al., 2019; Inoue, et al., 2020). These studies revealed that miRNAs are involved in ovarian function, and therefore we investigated the expression profiles of miRNAs in porcine ovaries with SLS and LLS than average litter sizes.

In this study, we selected a total of six sow ovaries with complete fertility records and used high-throughput sequencing technology combined with bioinformatic tools to investigate the miRNAs related to litter size. We characterized the function of miRNA-183 and it was shown to promote the proliferation of GCs in pig ovaries. This study not only provides a valuable transcriptional regulatory resource for understanding the mechanisms of pig ovarian function, but it can also reveal new clues for identifying the role of miRNAs in the determination of mammalian litter size.

## Materials and Methods

### Sample Preparation

In China, porcine reproductive and respiratory syndrome (PRRS) and porcine circovirus (PVC) diseases are the most important factors affecting litter size. Our experiment was conducted to guide the pig breeding industry and so we chose sows without these two diseases. In addition, the sows we selected were of the same age, body condition and were fed at the same nutritional level. Most importantly, they had no history of reproductive diseases. We collected the litter size records of the Yorkshire ✕ Danish Landrace binary hybrid sows from the Dahua breeding farm (*Guangxi Hanshiwei Food Co.*, *Ltd.*, from 2016 to 2018, which was consisted of 8,557 litters), and used SPSS 25.0 to perform a significance test on these data. After normal distribution conversion and inspection, it is found that the total litter size (12.9 ± 2.17) approximately obeyed the normal distribution. The critical value of 15% right tail probability was 14.7 heads per litter, and the critical value of 15% left tail probability was 9.3 heads per litter.

The animal welfare protocol and research plan were approved by the Animal Care Committee of Fuyang Normal University. The ovaries used in our study were obtained from sows (Yorkshire ✕ Danish Landrace binary hybrid pigs) 4 days after their fourth litter delivery. These were collected from a commercial pig farm (Nanning, Guangxi, China), which was negative for PRRSV and PCV. For RNA-seq, six ovaries were removed from each group with small (8.48 ± 0.53/litter; three samples) and large (16.19 ± 0.43/litter; three samples) litter sizes. The samples were snap-frozen in liquid nitrogen.

### Library Preparation and Illumina Sequencing

Total RNA was extracted from ovary tissue samples by using Trizol reagent (TaKaRa, Dalian, China). Gel electrophoresis, Agilent 2,100 and NanoDrop protocols were used to detect the integrity and concentration of RNA samples. Before constructing the RNA-seq libraries, the epicenter Ribo-ZeroTM Kit (Illumina, San Diego, CA, United States) was used to remove rRNA. Briefly, total RNA was purified by polyacryl-amide gel electrophoresis (PAGE) to enrich the sRNAs with lengths of 15–35 nt, then the sRNAs were ligated with adapters and amplified by RT-PCR. The amplification products were then separated by PAGE, and the transcriptome sequencing was performed on the HiSeq 2000 platform (Illumina, California, United States).

### Sequence Analysis

The process of filtering clean reads from the original data is as follows: 1) discard low-quality reads; 2) adjust the adaptor sequence; 3) remove sequences smaller than 13 bp. Then, the clean data was mapped to the pig reference genome (Sscrofa 11.1). The clean reads was searched for miRbase (www.mirbase.org) through BLAST. The unmapped reads was annotated and classified through Rfam (version 14.0; http://rfam.sanger.ac.uk) and BLAST in Genbank (www.ncbi.nlm.nih.gov) database. Finally, miRNA candidate genes were predicted by MIREAP (http://sourceforge.net/projects/mireap/) using the remaining unannotated siRNA sequences. Sequences located in the pig genome and folded into a typical hairpin structure with neighboring sequences were considered as potential new miRNAs. The new miRNAs were compared with the mature miRNAs of other mammals by BLAST.

### Differential Expression Analysis

To find the differentially expressed miRNAs between SLS and LLS samples, DESeq2 and Cuffdiff methods were performed using R statistical software’ Bioconductor. To find genes that were differentially expressed between SLS and LLS pigs, the identified miRNAs with a fold change of 2 or greater and *P*-adjusted < 0.05 were considered to be differentially expressed.

### Gene Ontology and Pathway Analysis

Gene ontology (GO) analysis (http://www.geneontology.org) was used to explore potential functions of miRNA. This was divided into three different aspects: biological process, cell composition and molecular function of the miRNA, which not only reduced the complexity but also highlighted the molecular function. Kyoto Encyclopedia of Genes and Genomes (KEGG) analysis (http://www.kegg.jp) was used to detect the participation of host genes in biological pathways.

### Target Gene Prediction

Based on the binding sites of miRNAs detected in mRNA sequences, the RNAhybrid (https://bibiserv.cebitec.uni-bielefeld.de/rnahybrid/) and MiRanda (http://cbio.mskcc.org/microrna_data/miRanda-aug2010.tar.gz) software were used to predict the interactions of miRNAs and mRNAs.

cDNA Synthesis, and Real-time qPCR.

Total RNA was reversely transcribed using a Hiscript III Reverse Transcriptase Kit (Vazyme, Nanjing, China). Real-time quantitative PCR (qPCR) was performed in triplicate using qPCR SYBR Green Master Mix (Vazyme, Nanjing, China) on a Bio-Rad CF96 system (Bio-Rad, United States), and U6 and GAPDH were used for normalization of the data.

### Cell Culture and Treatments

The isolation steps of pig ovarian GCs were performed as follows. The generic ovarian slaughterhouse tissues were digested with collagenase (Sigma, United States) in a 37°C water bath for about 1 hour. Then, the digested tissues were centrifuged at a low speed, and the supernatant was discarded, and the remaining tissue was filtered through a 200-mesh filter. The filtrate was washed three times with PBS, and finally the cells were resuspended with DMEM/F12 containing 1% penicillin/streptomycin. Then, the cells were seeded at a certain density on a cell culture plate at 37°C in an atmosphere of 5% CO_2_. In order to detect the transfection efficiency of the miR-138 mimic, miR-183 agomir (RiboBio, Guangzhou, China) was transfected into ovarian GCs when they were at 50% confluence using Lipofectamine 2000 (Invitrogen, United States).

### Measurement of E2 and P4

For measurement of the estradiol (E2) and progesterone (P4) levels in the culture supernatants, ELISA kits (Solarbio, Beijing, China) were used according to the manufacturer’s instructions. The inter- and intra-assay coefficients of variation were < 15 and < 10%, respectively for each kit. Each sample was measured in triplicate.

### CCK-8 and EdU Assays

The proliferation state of ovarian GCs was investigated with Cell Counting Kit-8 (CCK-8) (Tiandz, Beijing, China) and Cell-Light™ EdU Apollo^®^ 567 *In Vitro* Imaging Kit (RiboBio, Guangzhou, China), respectively. The manufacturer’s instructions were followed for all procedures.

### Western Blotting Analysis

Proteins were extracted from cell samples by using RIPA lysate buffer containing 1% PMSF (Solarbio, Beijing, China). The BCA Kit (Solarbio, Beijing, China) was used to measure the concentration of proteins in solution. The proteins were separated by 10% SDS- polyacrylamide gel (Bio-Rad, United States) electrophoresis and transferred to 0.22 μm nitrocellulose membranes (PALL, United States). After the membranes were incubated with primary antibodies specific for anti-cyclin D (cat. no. ab16663; dilution ratio = 1/200), anti-Bcl-2 (cat. no. ab692; dilution ratio = 1/200), anti-cyclin E (cat. no. ab33911; dilution ratio = 1/1000), anti-P27 (cat. no. ab32034; dilution ratio = 1/5000), anti-GAPDH (cat. no. ab8245; dilution ratio = 1/1000), and β-actin (cat. no. ab6276; dilution ratio = 1/5000) from Abcam (Cambridge, MA, United States) and secondary antibodies (dilution ratio = 1/5000; Solarbio, Beijing, China), the membranes were exposed with ECL Plus (Solarbio, Beijing, China). The images obtained were captured with the ChemiDoc XRS + system (Bio-Rad, United States).

### Flow Cytometry for the Cell Cycle Assay

GCs from pig ovaries were treated with miR-183 agomir in 6-well plates. After 24 h, the cells were treated with the Cell Cycle Assay Kit (Multisciences, Hangzhou, China), washed with PBS buffer and centrifuged to remove the supernatants. Then permeabilization and DNA stain solutions were added to the re-suspended cells and mixed by vortex shaking. After incubation in the dark for 30 min, the cells were analyzed by Flow Cytometry (FACS Canto™ II, BD BioSciences, United States).

### Statistical Analyses

Results are shown as means ± standard error of the means (SEM) for at least three independent experiments. SPSS 25.0 software (Chicago, United States) was used to perform a significance test on these data. *p*-values were calculated by Student’s *t*-test. *p* < 0.05 was considered as statistically significant for RNA expression, protein expression, CCK-8 and EdU assays and measurement of E2 and P4.

## Results

### Identification of miRNAs Expressed in Pig Ovary

**FIGURE 1:**
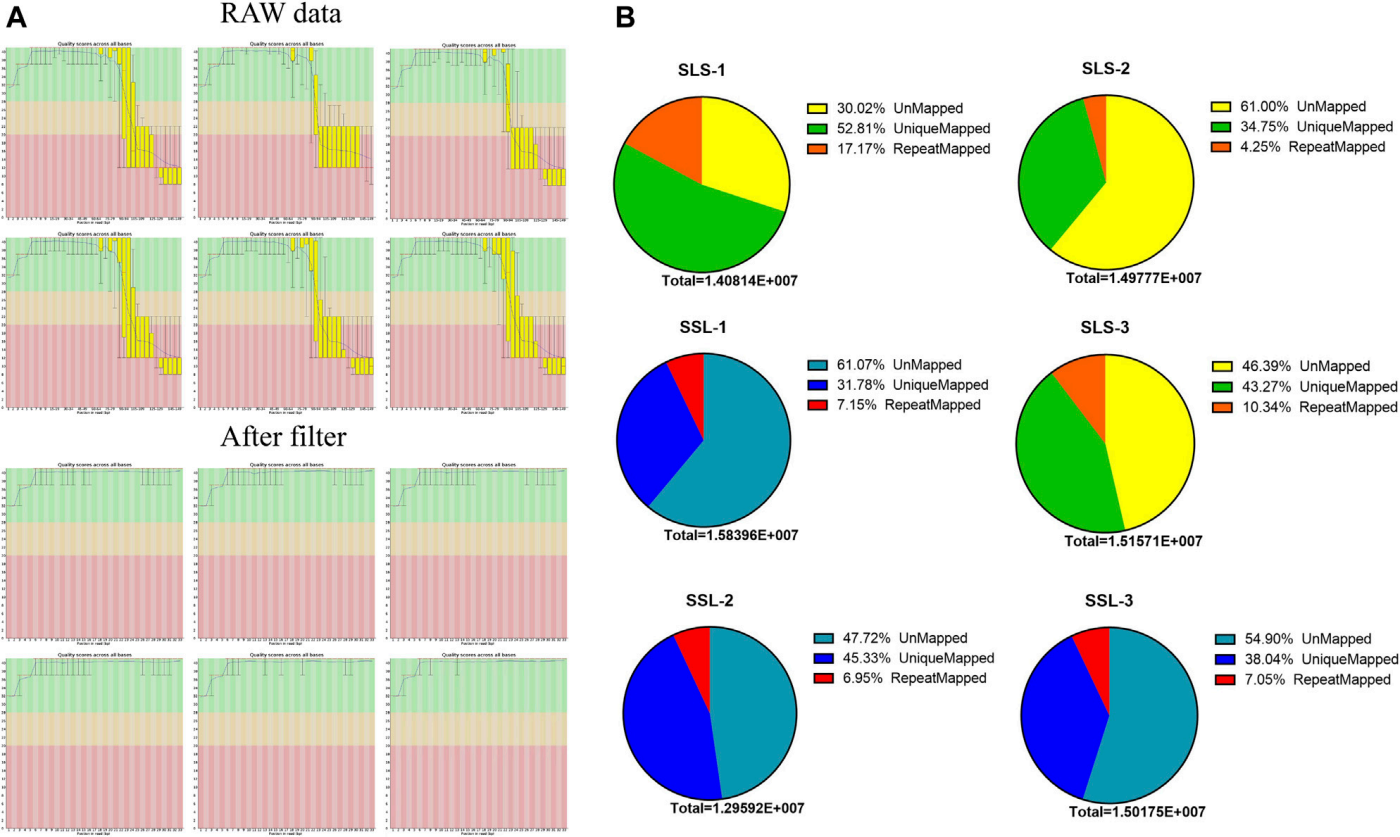
MicroRNA data filtering and mapping of the swine genome. **(A)** Data quality control, through the data scores and filtering low-quality data of microRNA-seq for subsequent analysis. **(B)** The microRNA data mapped rates of high (LLS) and low (SLS) fertility swines (*n* = 3).

In order to discover the role that miRNAs play in the determination of pig litter size, six sow ovary samples with complete fertility records were selected to perform high-throughput sequencing. After sequencing, Fast-QC (http://www.bioinformatics.babraham.ac.uk) was used to make an overall evaluation of the original data. As shown in Figure 1A, the Q30 values of the LLS pigs in the tested samples were: 92.56, 92.40 and 82.21%, and the SLS pigs were 90.21, 92.46 and 78.43% (Figure 1A). It was worth mentioning that the Q30 values of two samples were low (only about 80%), but the mapped reads and unique mapped reads were not less than other samples, indicating that the data quality of this sequencing could be used for subsequent research (Table 1). The results of 64.5 million (M) and 61.6 M total reads were obtained from the ovarian libraries of SLS and LLS pigs, respectively. After removing the low quality and adaptor sequences, a total of 44.2 and 43.8 M clean reads, respectively, were obtained (Figure 1B; Table 1). The length of miRNA-seq reads ranged from 20 to 24 nucleotides; the analysis revealed that a majority of reads were 21 and 23 nt (Supplementary Figure S1).

**TABLE 1:** MicroRNA-seq data that were used for quality control.

Sample name	Total reads beafore	Total reads after	Mapped reads	Unique mapped reads	Unique mapped rate
SLS1	21896512	14,081,415	9,854,086	7,436,865	0.528
SLS2	20,516,344	14,977,666	5,841,554	5,204,463	0.347
SLS3	22,085,862	15,157,118	8,125,429	6,558,056	0.433
LLS1	22,021,340	15,839,580	6,165,721	5,033,630	0.318
LLS2	18,484,476	12,959,228	6,775,070	5,874,644	0.453
LLS3	21,115,843	15,017,466	6,772,128	5,713,064	0.38

### Differential Expression Analysis of MiRNAs

**FIGURE 2:**
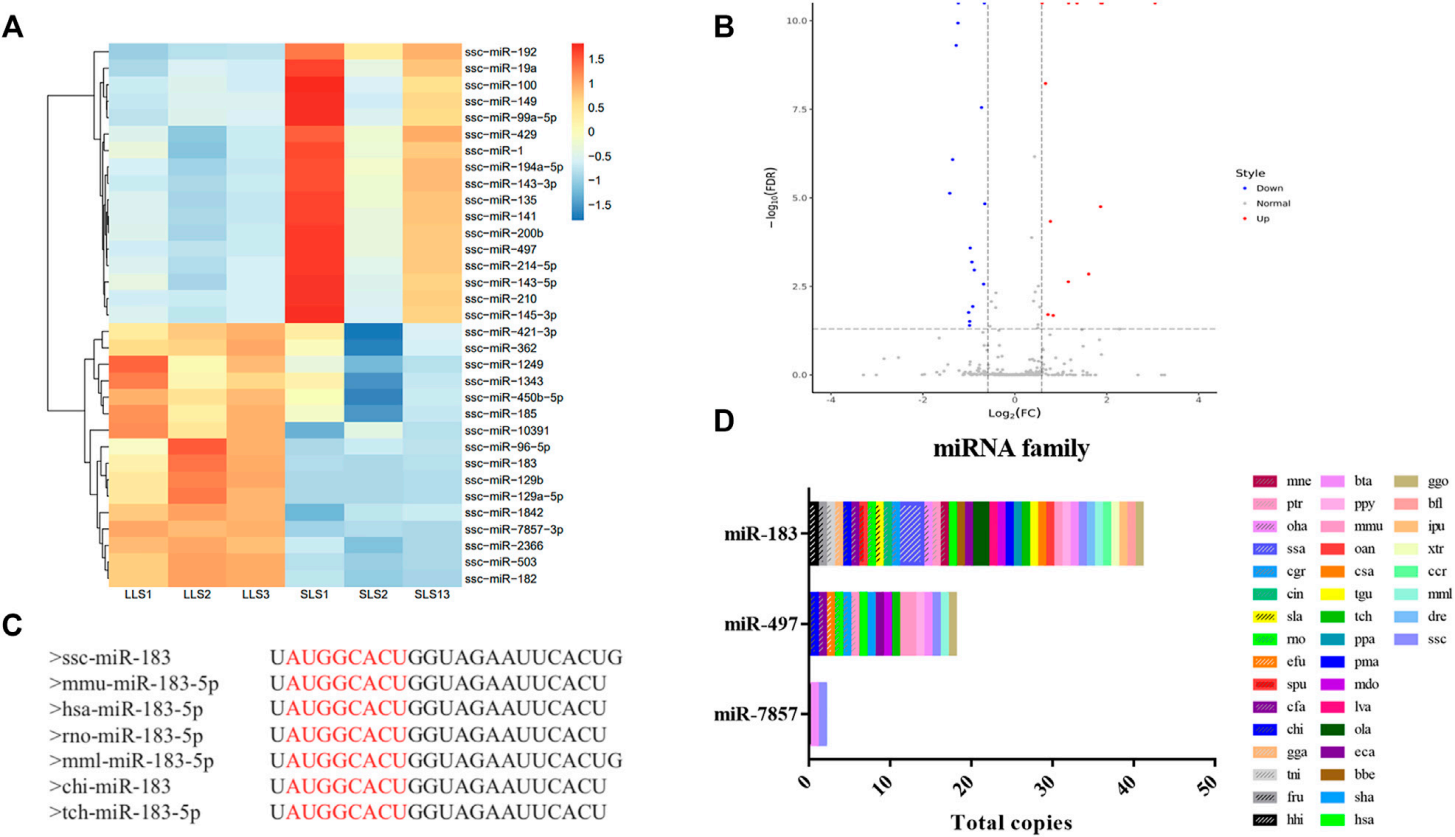
Differential expression and homology analysis of candidate microRNAs. **(A,B)** A total of 33 differentially expressed miRNAs were screened in the two groups, as shown in the heatmap and volcano map, LLS vs SLS 16 miRNAs were up-regulated and 17 miRNAs were down-regulated. **(C)** The conservative sequence of mir-183 is shown as “AUGGCACU”. **(D)** The family analysis of miR-183, miR-7857-3 and miR-497, of which miR-183 and miR-497 were widely present in multiple species.

A total of 411 miRNAs were identified, and 17 miRNAs were significantly down-regulated and 16 miRNAs were up-regulated when comparing LLS pigs to SLS pigs, respectively (Figures 2A,B, Supplementary Table S1). It can be seen that miR-183-5p is one of the most significant differential expressions. Its expression level in the LLS group was 1,512 (TPM) compared to 522 in the SLS group (TPM, Supplementary Table S2). It can also be seen that miR-183-5p is conserved in more than 35 species including humans, mice and pigs **(** Figure 2C, Figure 2D).

To explore the possible biological functions of miRNAs identified in SLS and LLS porcine ovarian tissues, GO analysis was performed (Supplementary Figure S2). The results showed that these miRNAs were significantly enriched by 20 biological processes, including steroid biosynthesis process, lipid metabolism process, ovarian follicle development, granular cell differentiatio. In order to better understand the molecular regulation of DEGs, we performed KEGG pathway analysis (Supplementary Figure S3). The hippo signaling pathway was the most significant (path: 04390). From the three enrichment pathways of steroid biosynthesis (path: 00100), ovarian steroid production (path: 04913) and steroid hormone biosynthesis (path: 00140), it could be seen that the synthesis and metabolism of steroids play an important role in the ovaries of pigs.

**FIGURE 3:**
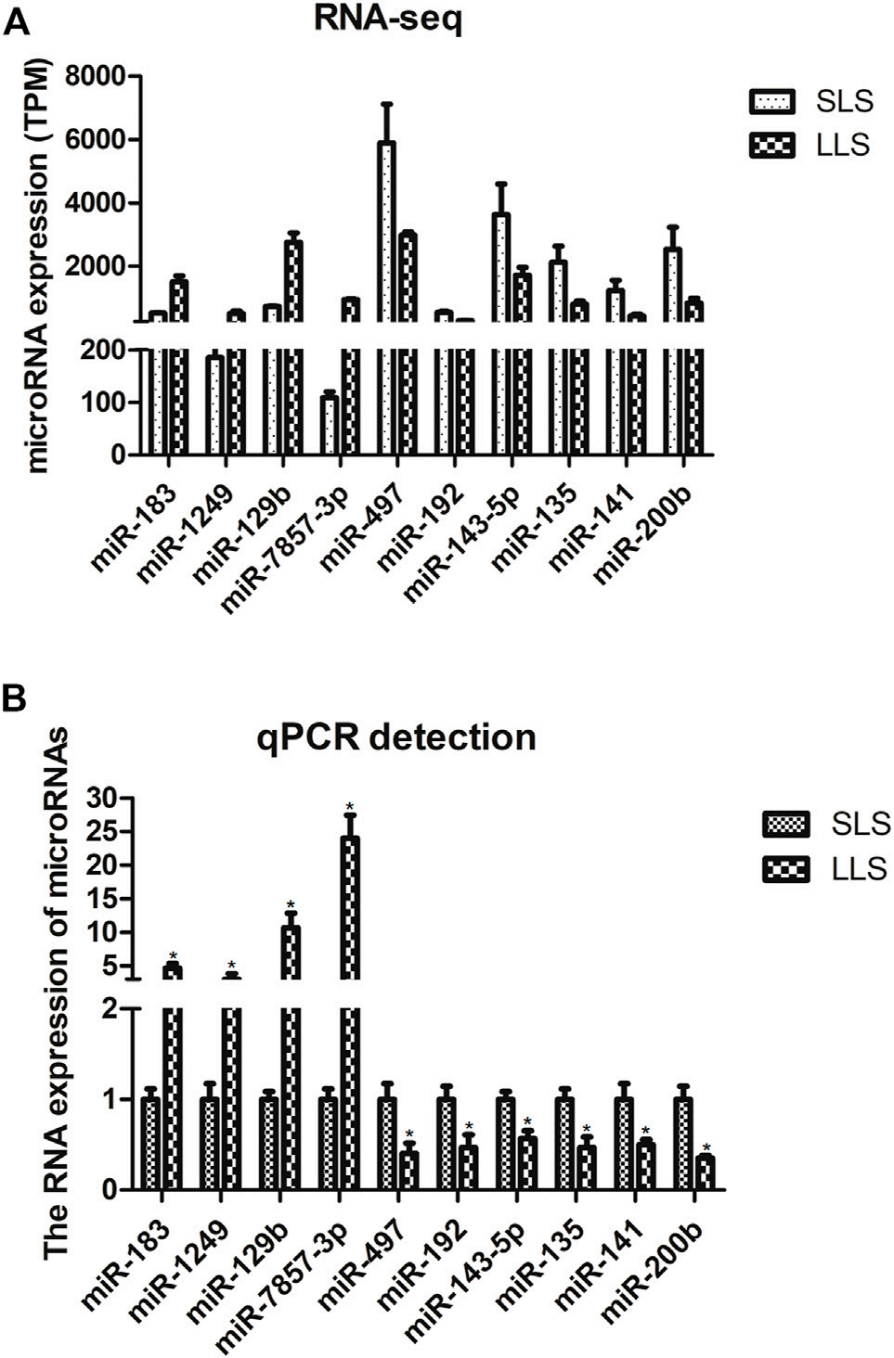
Validation of candidate differentially expressed microRNAs by quantitative PCR (*, *p* < 0.05; *n* = 3).

We confirmed the differences in the abundance of certain miRNAs when comparing SLS and LLS tissue samples by using qPCR. We randomly selected 10 differentially expressed miRNAs and amplified these using qPCR primers (Figure 3). Our qPCR analysis found the presumed patterns of up- and down-regulation of these 10 miRNAs to be remarkably similar in both types of analysis (Figure 3), suggesting that miRNA-Seq data provided reliable information about the relative abundance of miRNAs. Among these miRNAs, miR-183 is conserved in 38 species and has high expression level in the ovaries of LLS sows. This suggests that it may play a potential role in regulating porcine litter size.

### Effect of MiR-183 on Gene Expression of Ovarian Function

**FIGURE 4:**
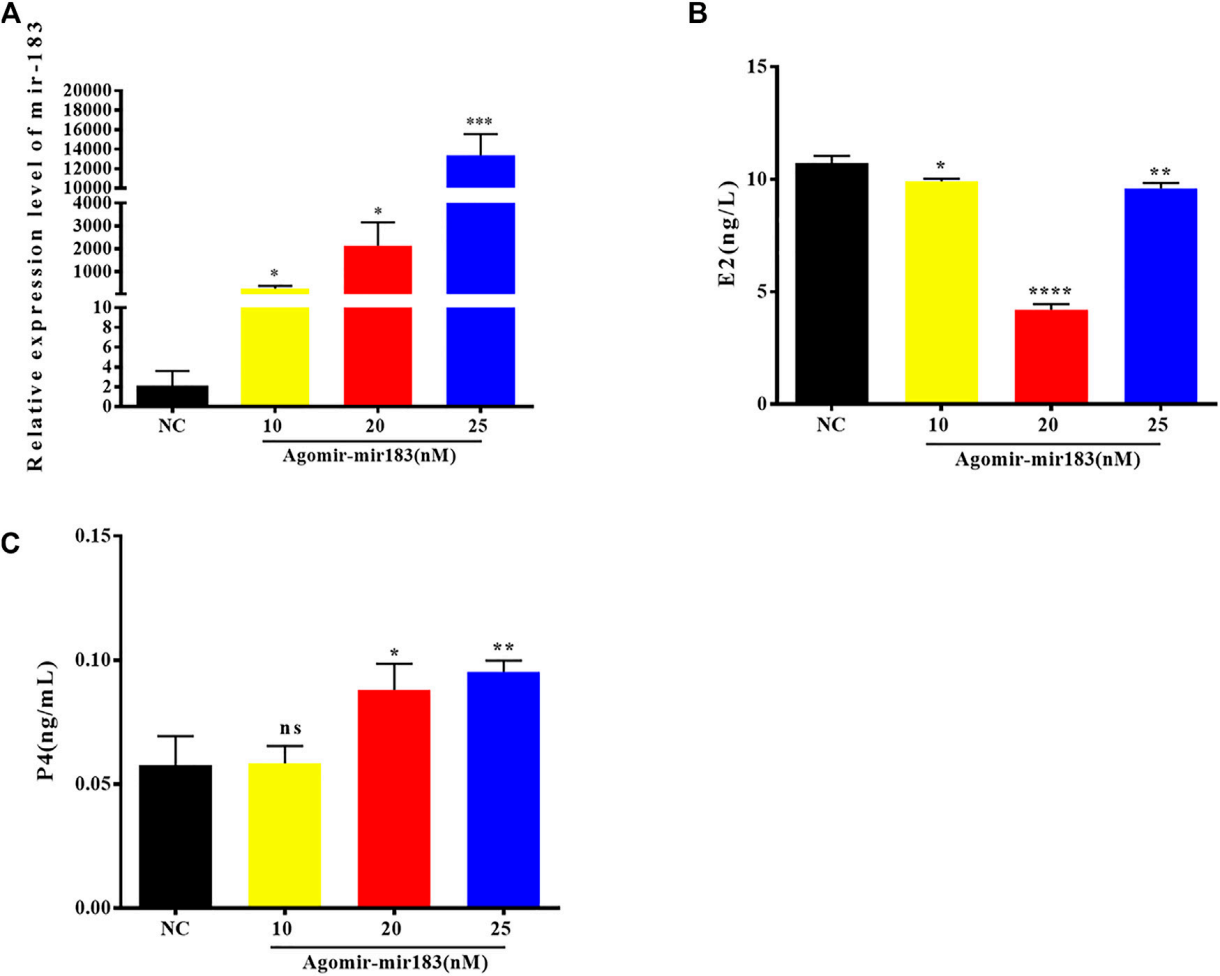
Overexpression efficiency of miR-183 **(A)** Assessment of overexpression efficiency by qPCR. **(B, C)** Compared with NC, 20 nM miR-183 agomir significantly reduced the synthesis of E2 and promoted the synthesis of P4 (NS: no significant difference; *, *p* < 0.05; **, *p* < 0.01; ***, *p* < 0.001, *n* = 3).

In order to understand the effect of miR-183 on the function of ovarian GCs, the expression changes of key functional genes in GCs after overexpression of miR-183 were examined. After transfection of miR-183 agomir **(**a mimic), in porcine GCs, the overexpression efficiency was measured by qPCR. The cells were collected after transfection with three different concentrations of agomir for 24 h. The results showed that the concentrations of agomir at 10, 20 and 25 nM were able to stably overexpress miR-183, while the transfection concentration of 20 nM could significantly reduce the synthesis of E2 and promote the synthesis of P4. Therefore, for the next round of functional verification, we chose a transfection concentration of 20 nM to overexpress miR-183 in GCs (Figure 4).

**FIGURE 5:**
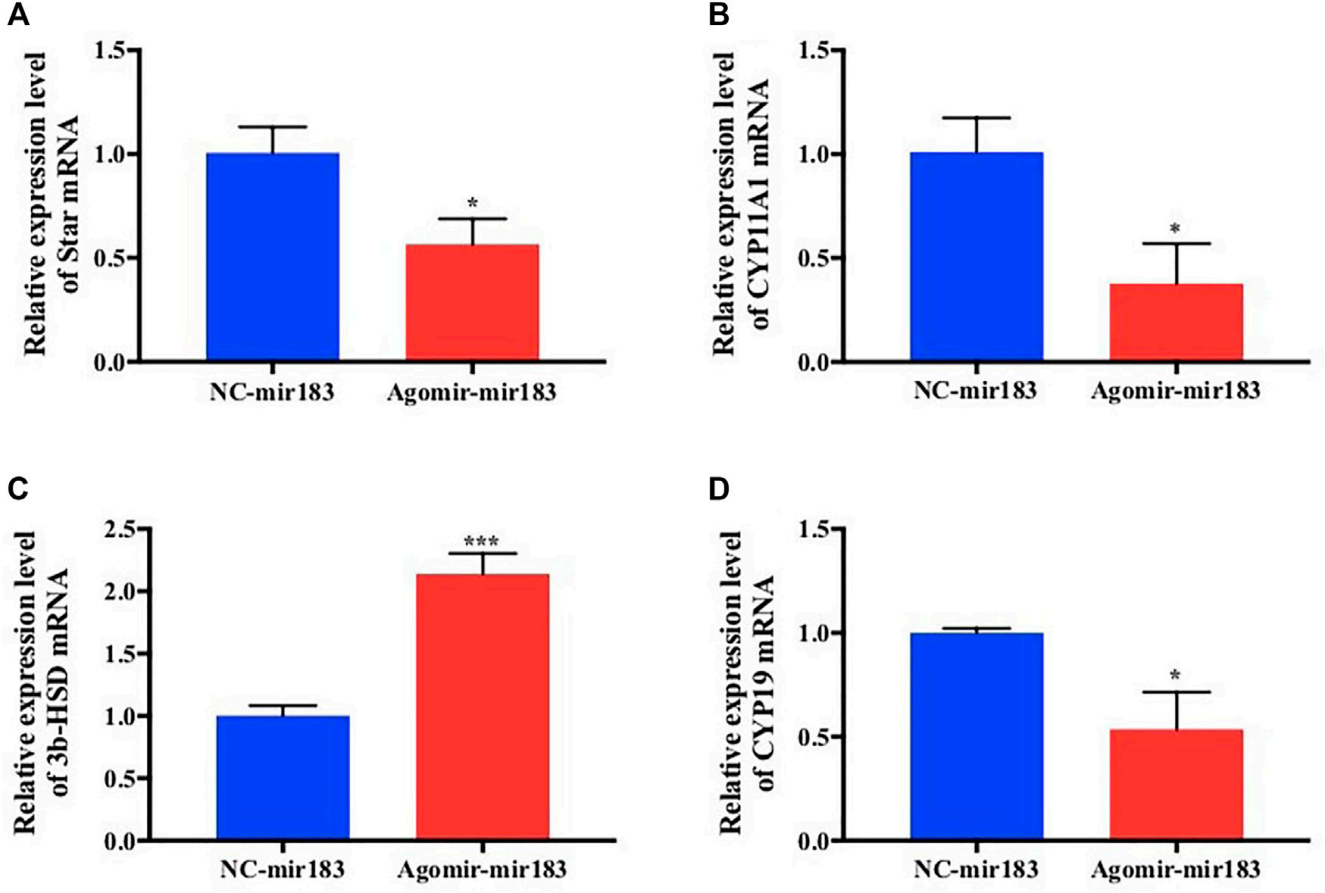
Effect of overexpression of miR-183 on granulocyte cytokine synthesis **(A–D)** After mir-183 was overexpressed in granulosa cells, the mRNA expression levels of Star, CYP11A1, 3β-HSD and CYP19 were measuerd by qPCR assay (*, *p* < 0.05; ***, *p* < 0.001, *n* = 3).

After treatment with 20 nM miR-183 agomir for 24 h, the GCs were collected and RNA was extracted, and measured by qPCR. It was found that the expression of hormone synthesis related genes Star, Cyp11a1 and CYP19 were decreased significantly (*p* < 0.05), but the expression levels of 3β-HSD were markedly increased (*p* < 0.0001; Figure 5).

### Effect of MiR-183 on Cell Proliferation and Apoptosis

**FIGURE 6:**
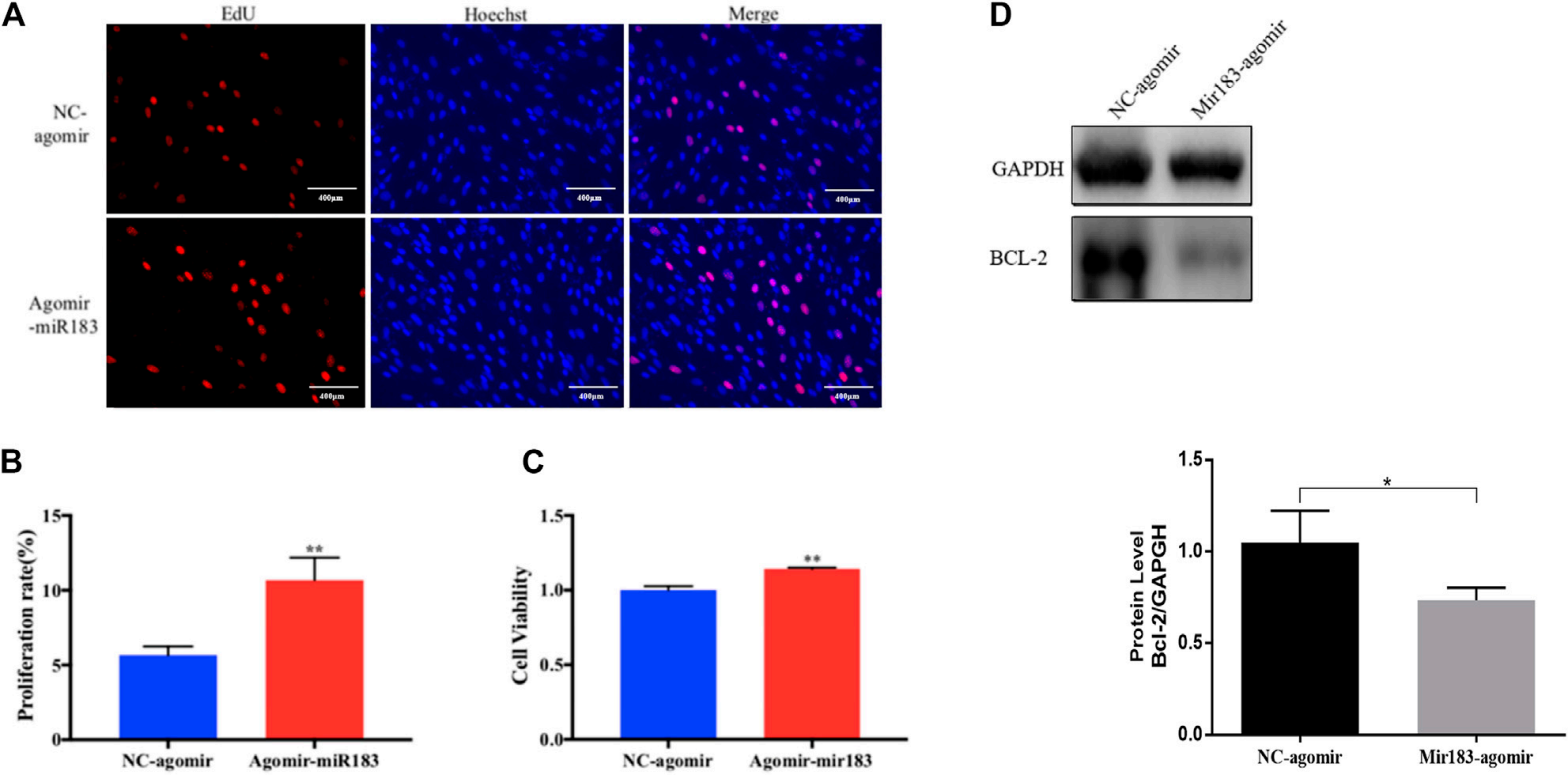
Effect of mir-183 on the proliferation and apoptosis of granulosa cells. **(A)** EdU staining test was used to detect the proliferative capacity of granulosa cells. Red represents newly-produced EdU-positive cells and blue represents the nuclei (Hoechst); the scale is 100 μm. **(B)** Quantification of EdU-positive cells, *n* = 3. **(C)** CCK-8 assay to assess the viability of granulosa cells at 24 h, *n* = 5. **(D)** The amount of apoptosis protein Bcl-2 expression. GAPDH protein was used as an internal reference. The results are expressed as mean ± standard error, *n* = 3; NS: no significant difference; *, *p* < 0.05; **, *p* < 0.01.

**FIGURE 7:**
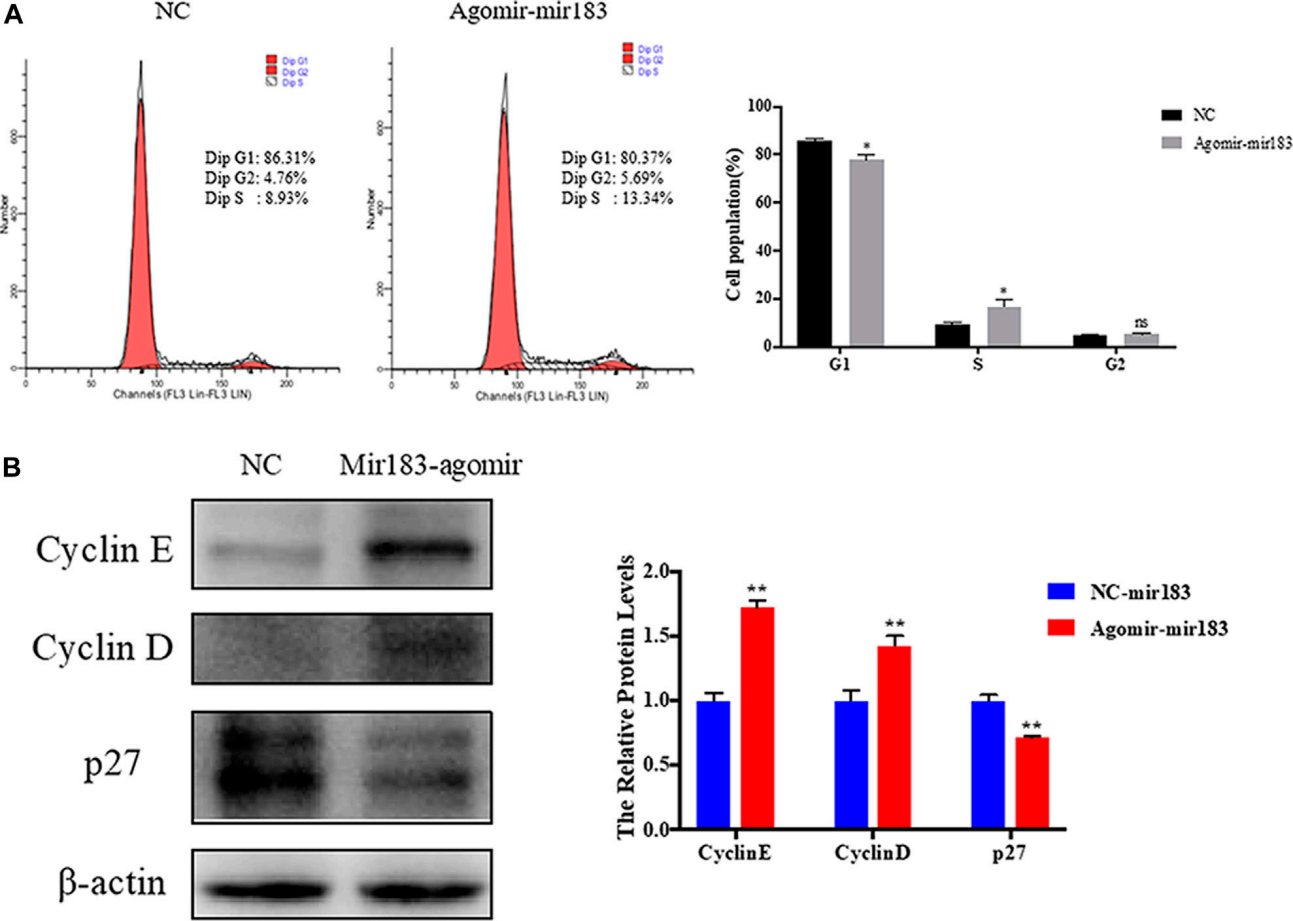
Regulation of miR-183 in cell cycle of granulosa cells. **(A)** The cell cycle of granulosa cells after transfection of miR-183; **(B)** Cyclin E, Cyclin D and p27 protein expression and quantification. β-actin protein was used as an internal reference.

To further clarify the effect of miR-183 on the proliferation and apoptosis of ovarian GCs, miR-183 was overexpressed in these cells. The effect of miR-183 on the proliferation ability of GCs was assessed by EdU staining. Compared with the control group, miR-183 agomir significantly increased the number of EdU GCs positive cells (Figures 6A,B). Similarly, miR-183 could significantly increase cell viability as detected with the CCK-8 kit (*p* < 0.05, Figure 6C). In order to detect the effect of miR-183 on apoptosis, we treated GCs with 20 nM miR-183 agomir for 24 h. After the GCs were collected, the protein expression levels of the anti-apoptotic protein, Bcl-2, was measured by using Western blot. It was found that there was a tendency to decrease the expression levels between control and treatment groups (*p* < 0.05, Figure 6D). Moreover, miR-183 significantly increased the number of cells in the S phase and this was shown by flow cytometry (Figure 7A). It was also found that miR-183 overexpression increased the expression of cyclins D and E, and decreased the expression of p27 at the protein level (Figure 7B).

## Discussion

The ovary is one of the important reproductive organs of mammals, and it regulates follicular development and hormone secretion. Previous studies have shown that mirRNAs can regulate follicular development (Du, et al., 2019; Yang, et al., 2019; Zhang, et al., 2019; Ma, et al., 2020; Li, et al., 2021). In this study, we used high-throughput sequencing to investigate miRNA expression in the ovaries of differing litter sizes. This study will contribute to understand the role of miRNA in regulating porcine reproductive system.

The number of live births in litters affects the profitability of the pig industry. The mammalian ovary plays an important role in the reproduction process, including ovulation and hormone secretion, and these are closely related to the regulation of specific microRNAs. Previous studies have found that the ovaries of Yorkshire pigs with LLS and SLS can express a large number of miRNAs, and a total of 37 differentially expressed miRNAs were obtained (Huang, et al., 2016; Ma, et al., 2018; Xu, et al., 2019; Bakoev, et al., 2020; Zheng, et al., 2020). These studies showed that miR-224, miR-99a and miR-183 were the miRNAs with the most significant differences and these might affect litter size. In this study, we collected the litter size records of the Yorkshire ✕ Danish Landrace binary hybrid sows prior to sequencing the samples. Compared with previous studies, the samples we selected were more representative of the swine industry in China. Furthermore, previous studies did not study the function of candidate miRNAs after finding differentially expressed molecules. This study was able to demonstrate that miR-183 promoted the proliferation of GCs of pig ovary.

MiR-183 belongs to the highly conserved miR-183-96-182 cluster and has been reported to be related to female fertility (Mohammed et al., 2019). It is known that members of the miR-183-96-182 cluster could target the 3′-UTR of FOXO1 mRNA (Leung, et al., 2015; Ichiyama, et al., 2016), which is an important transcription factor for follicle stimulating hormone response genes in mammalian ovarian GCs. Studies have shown that FOXO1 and miR-183-96-182 clusters are related to the development of bovine ovarian follicles (Gebremedhn, et al., 2016; Mohammed, et al., 2017). Bioinformatics analysis indicated that miR-183 may be related to gene transcription, especially PI3K-Akt signaling. Genome-wide analysis showed that the follicle differentiation of pig ovary is closely related to the expression of PI3K-Akt pathway-related genes. After the growth of pig ovarian GCs are stimulated, and PI3K-Akt activity is significantly inhibited, this in turn, affects the secretion of 17β-estrogen from ovarian GCs (Zhang et al., 2018). Furthermore, miR-183 is highly expressed in ovarian cancer cells (Li, et al., 2012; Chen, et al., 2016; Qi, et al., 2019), and the down-regulation of miR-183 can inhibit cell proliferation and induce apoptosis of SMAD family member 4 (Zhou, et al., 2019). In this study, 411 miRNAs were identified, and we further characterized one of the most significantly differentially expressed miRNAs-miR-183. In addition, miR-183 has been shown to promote the proliferation of porcine ovarian GCs and affect the synthesis of E2 and P4. These results suggest that miR-183 may participate in the regulation of biological processes of pig prolificacy by affecting the cellular proliferation. Overall, by investigating multiple ncRNAs it may be possible to develop treatments for reproductive capacity of sows in the future.

## Conclusion

Transcriptome sequencing was used to map the expression of miRNAs in pig ovaries. Comparing pig ovaries from sows with LLS and SLS, a large number of miRNAs were found, and some had different abundances. miR-183 is one of the highly expressed differential miRNA in LLS pigs, and we characterized and evaluated its function. We found that miR-183 promotes the proliferation of ovarian GCs and regulated the synthesis of E2 and P4. This study has increased the understanding of the genetic mechanism of pig ovarian function and may provide new molecular targets for the continued successful breeding of pigs in China.

## Data Availability

The datasets presented in this study can be found in online repositories. The names of the repository/repositories and accession number(s) can be found in the article/Supplementary Material.
