# Supplementary figures and images for: Discovery of Differentially Expressed MicroRNAs in Porcine Ovaries With Smaller and Larger Litter Size
Source: Front Genet. 2022 Feb 9;13:762124. doi: 10.3389/fgene.2022.762124 (PMC8864311; doi:10.3389/fgene.2022.762124)

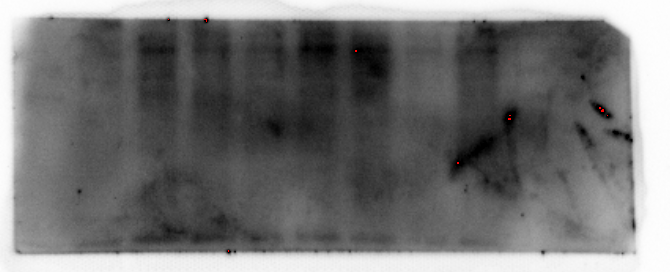

Supplement: Supplementary file 3 [file Image3.JPEG]

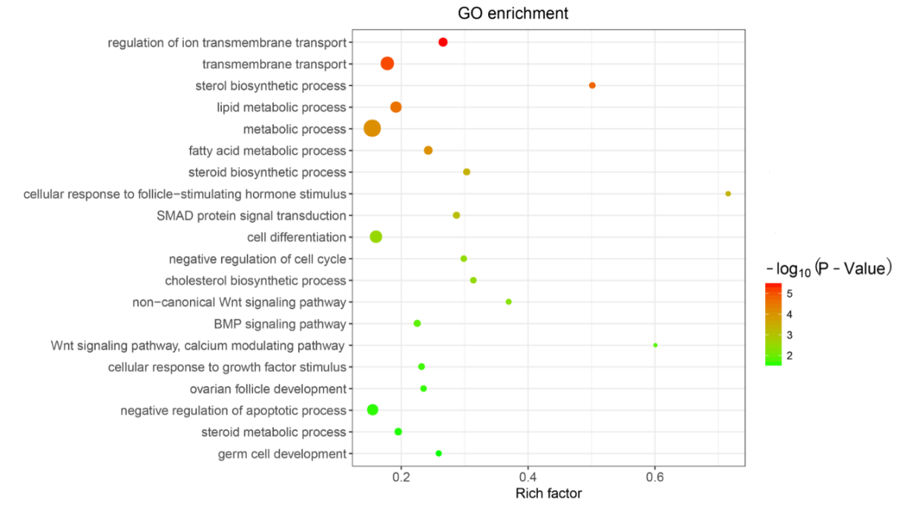

Supplement: Supplementary file 6 [file Image14.TIF]

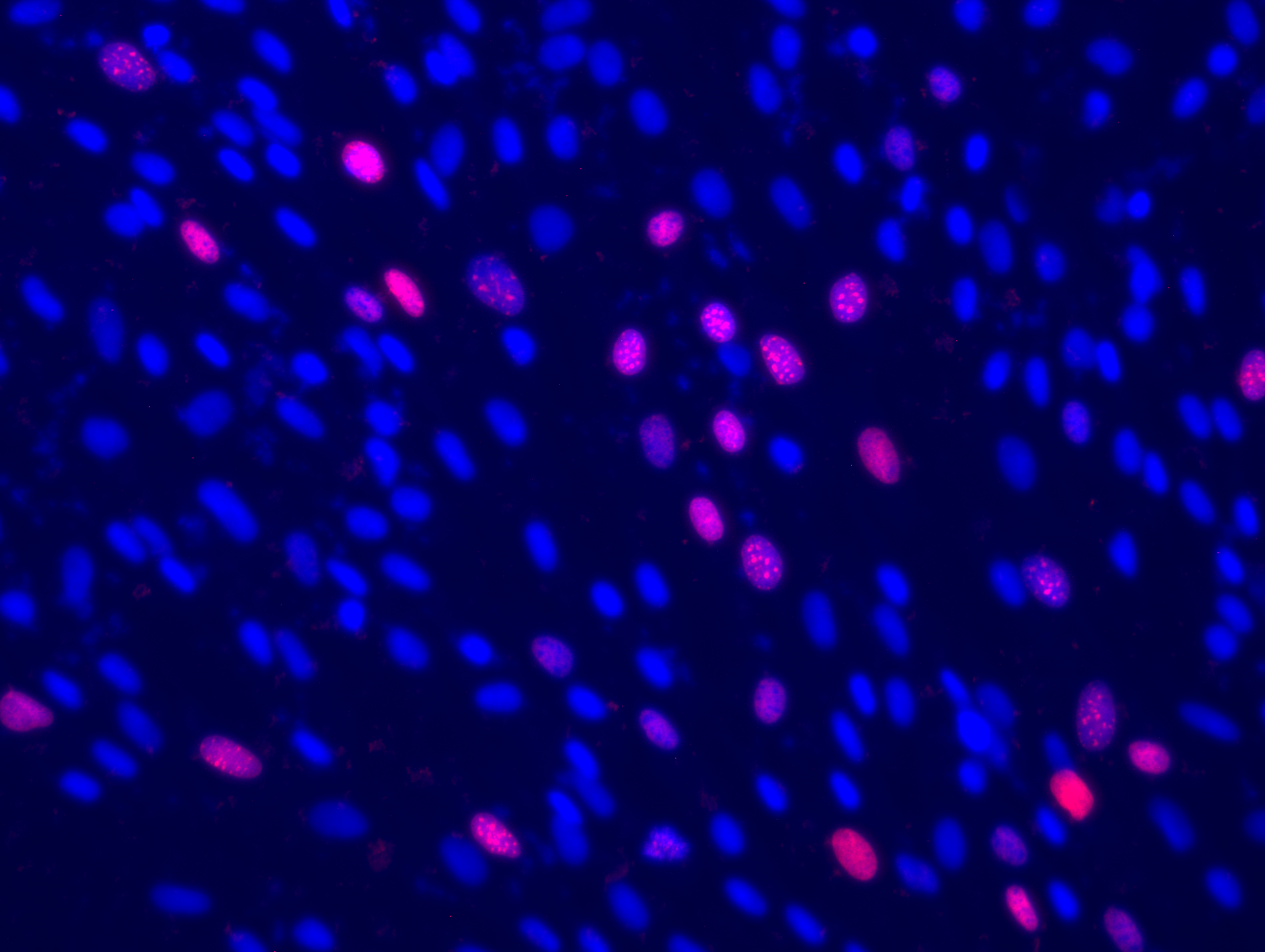

Supplement: Supplementary file 7 [file Image9.TIF]

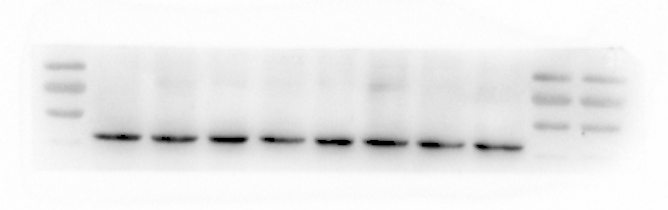

Supplement: Supplementary file 8 [file Image1.JPEG]

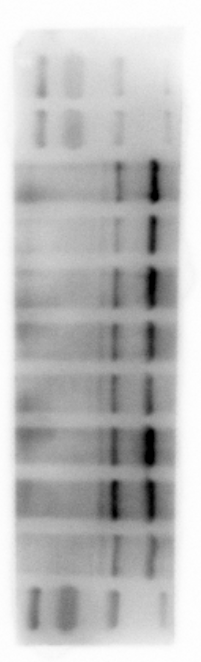

Supplement: Supplementary file 9 [file Image4.JPEG]

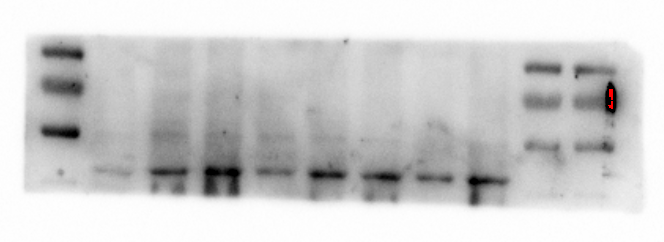

Supplement: Supplementary file 10 [file Image2.JPEG]

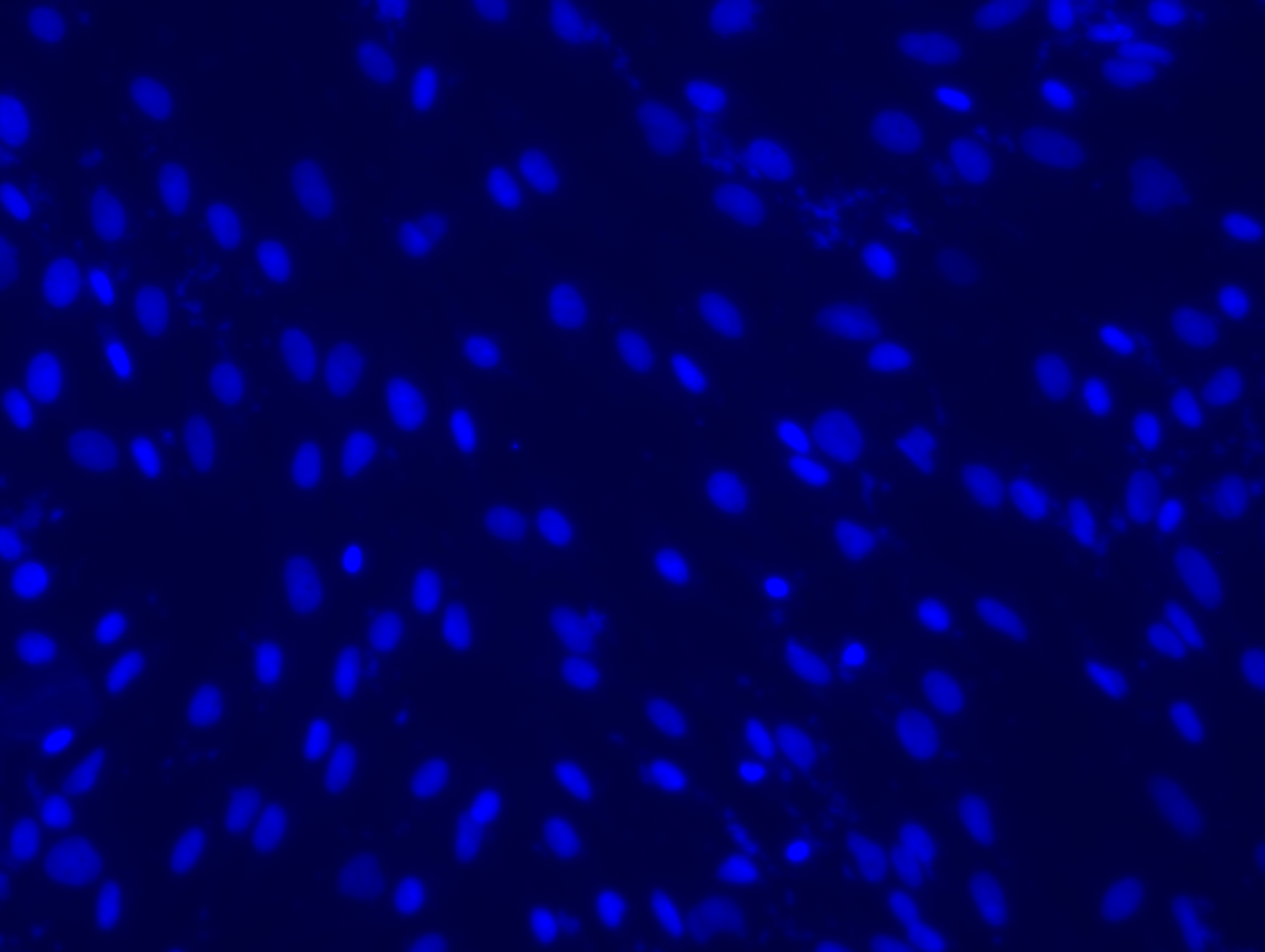

Supplement: Supplementary file 11 [file Image11.TIF]

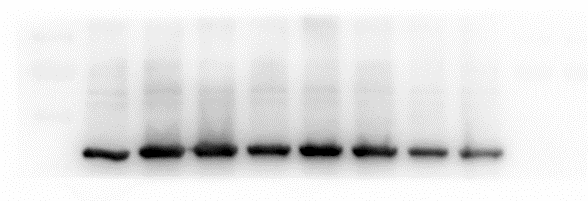

Supplement: Supplementary file 12 [file Image5.JPEG]

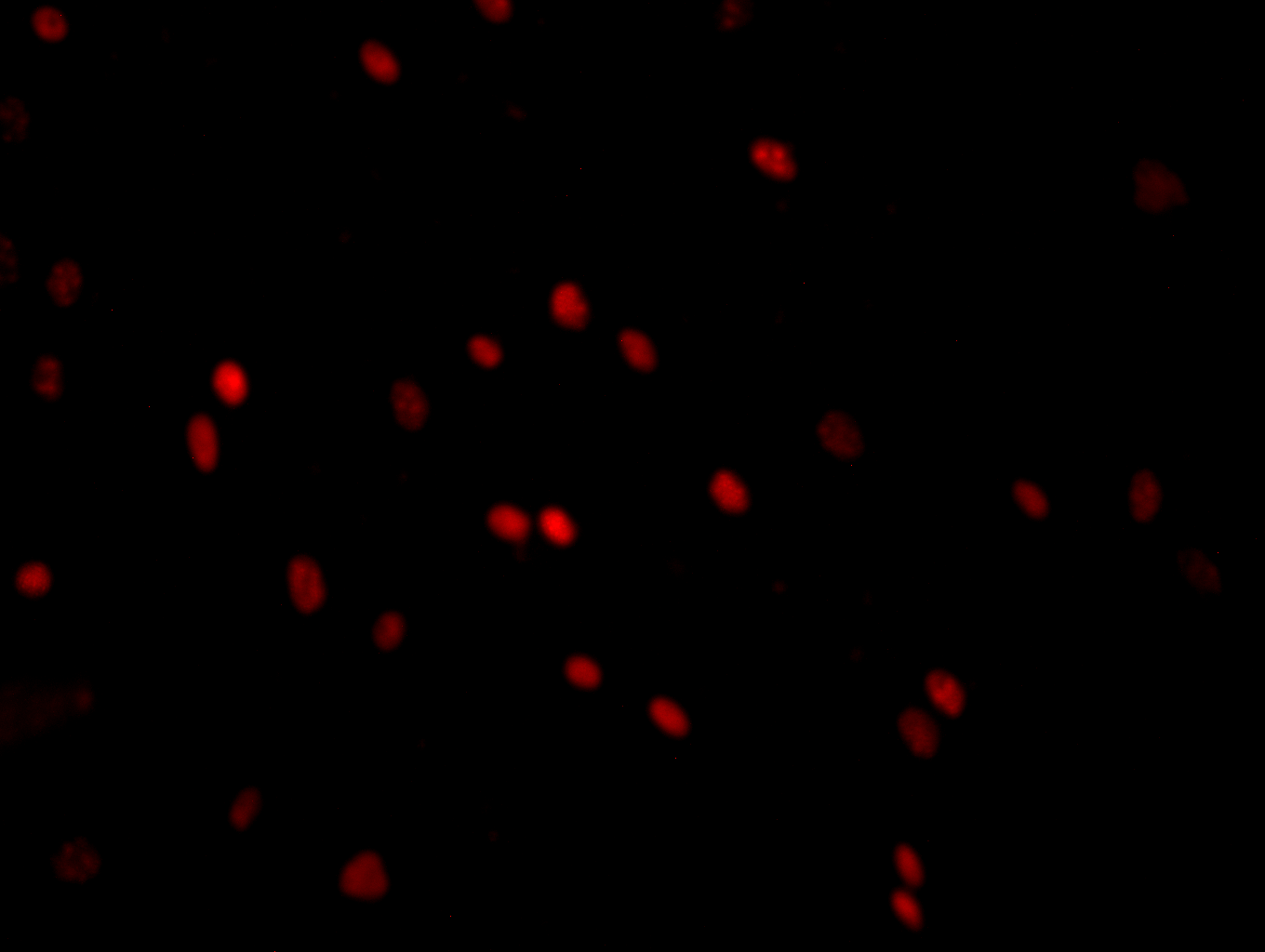

Supplement: Supplementary file 13 [file Image10.TIF]

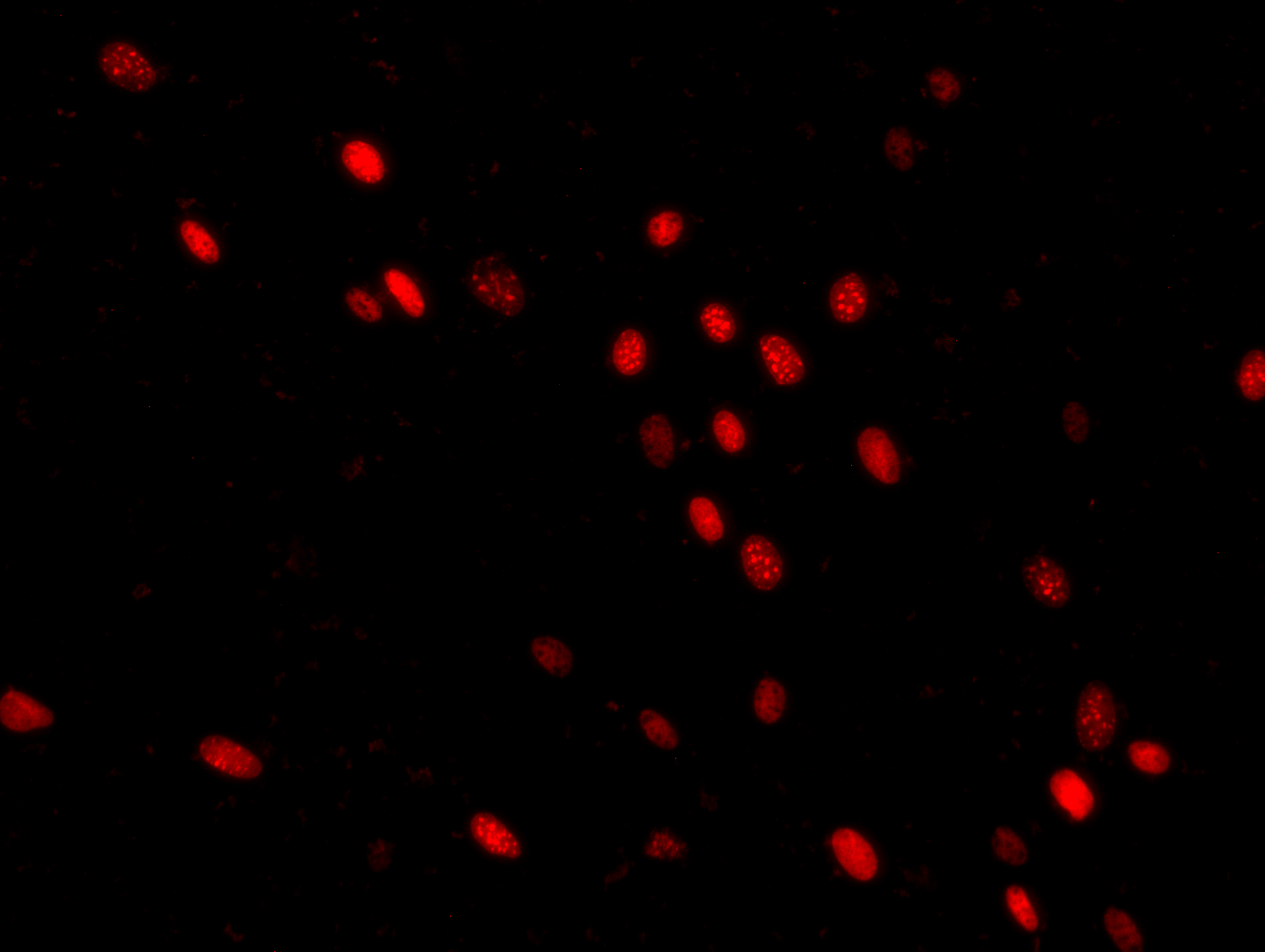

Supplement: Supplementary file 14 [file Image7.TIF]

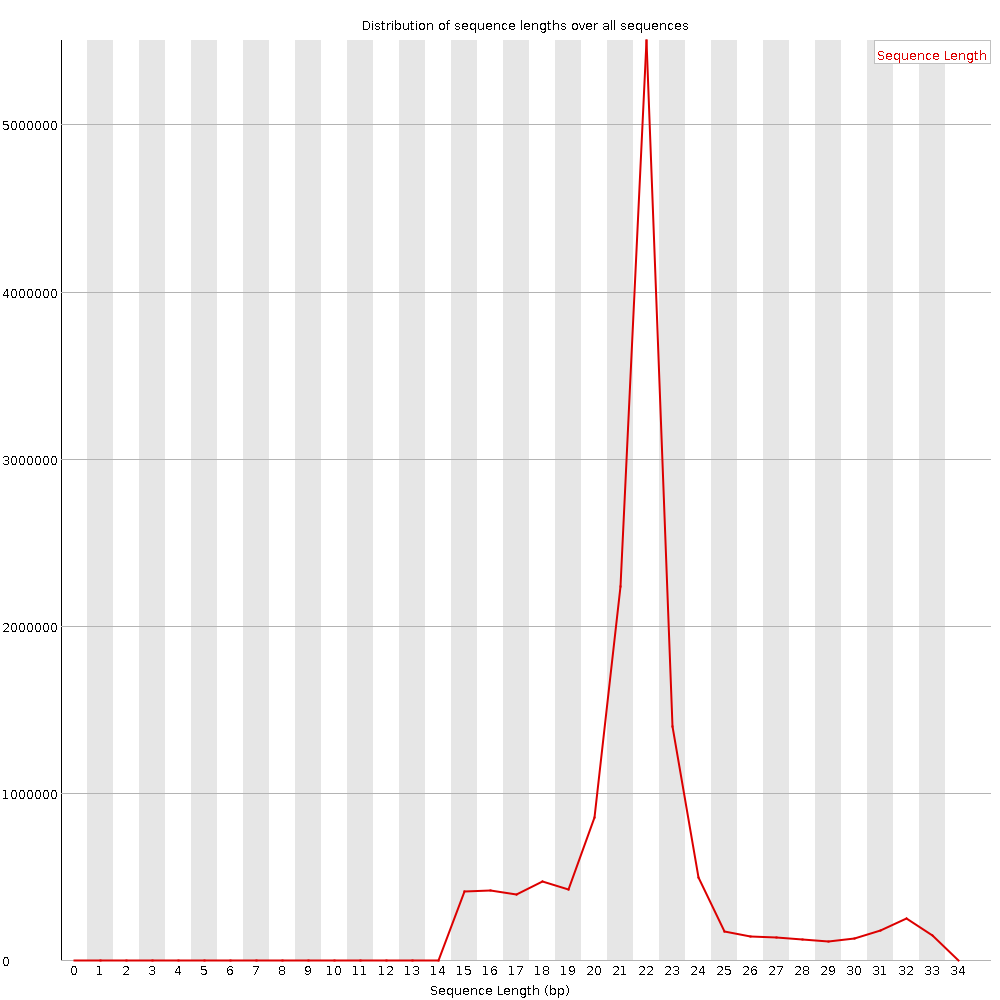

Supplement: Supplementary file 16 [file Image13.PNG]

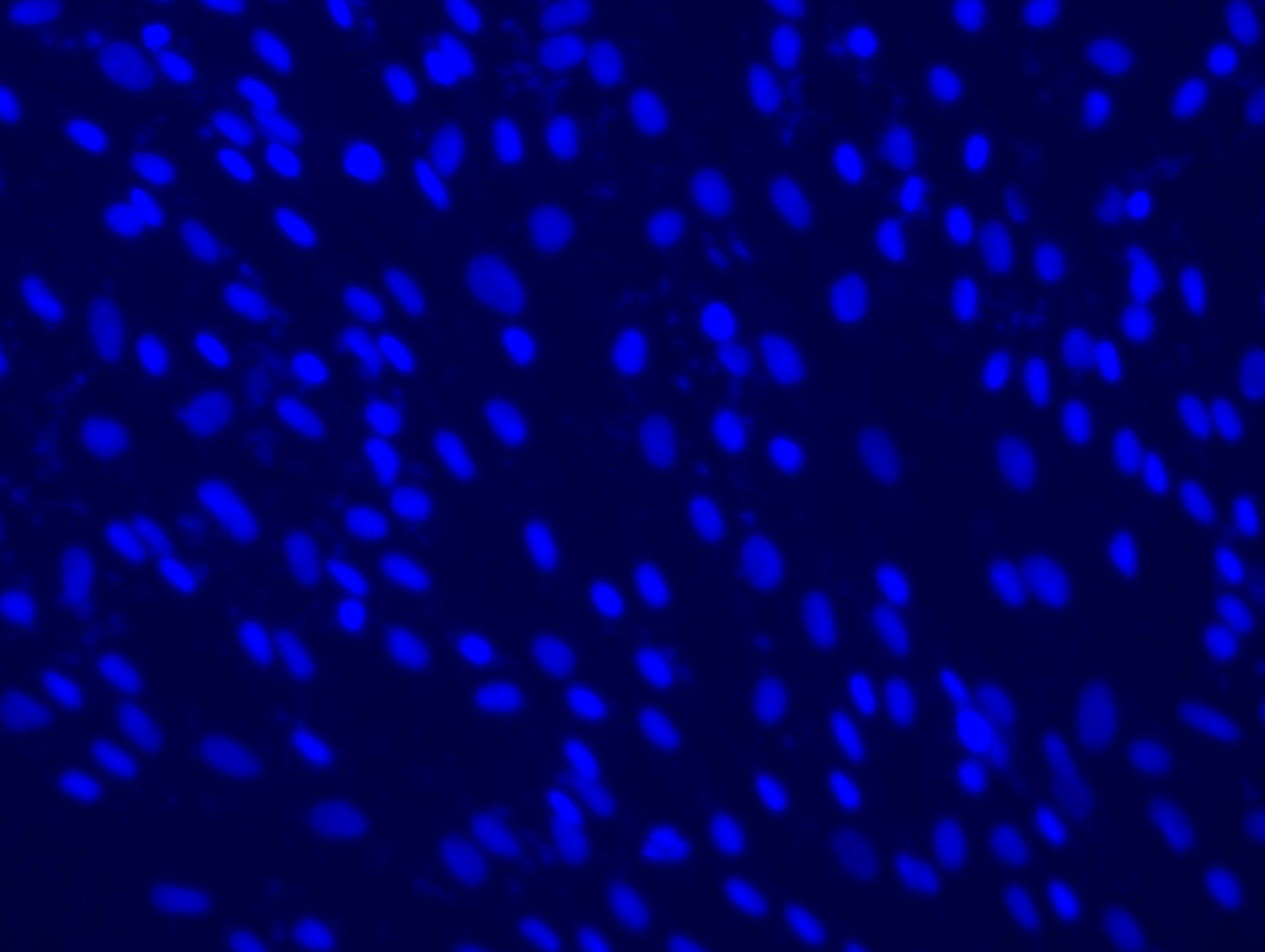

Supplement: Supplementary file 24 [file Image8.TIF]

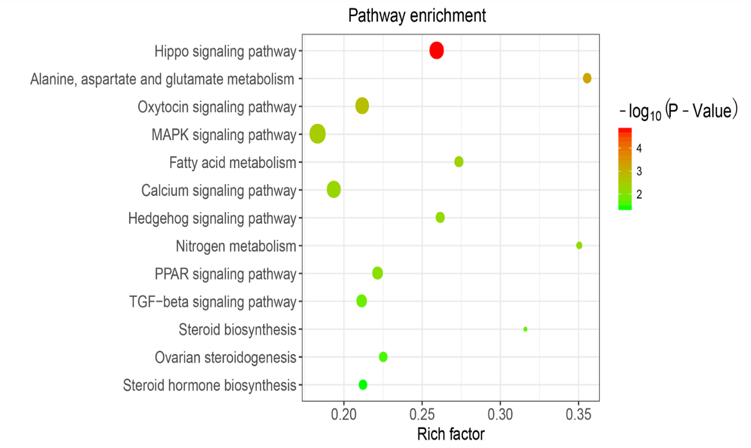

Supplement: Supplementary file 25 [file Image15.TIF]

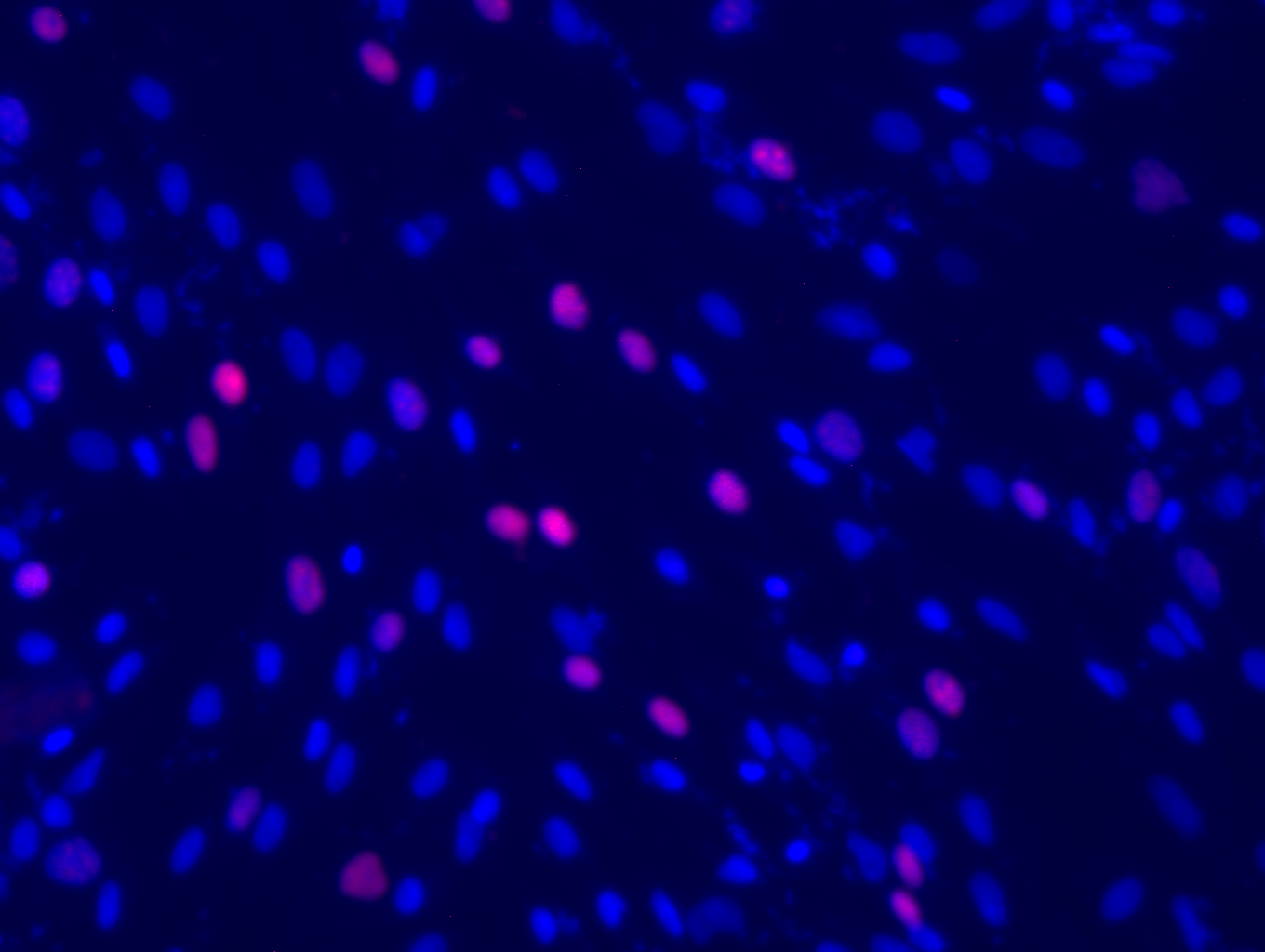

Supplement: Supplementary file 26 [file Image12.TIF]

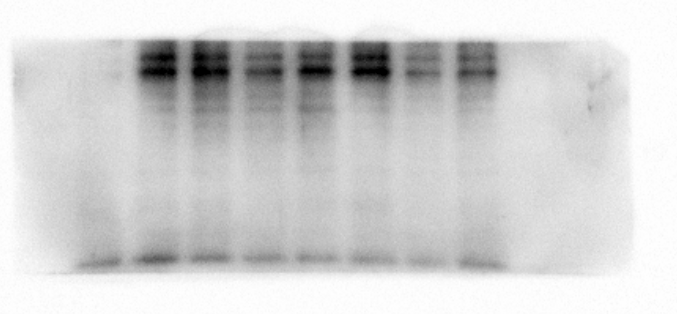

Supplement: Supplementary file 30 [file Image6.JPEG]
