# Supplementary material for: Discovery of Differentially Expressed MicroRNAs in Porcine Ovaries With Smaller and Larger Litter Size
Source: Front Genet. 2022 Feb 9;13:762124. doi: 10.3389/fgene.2022.762124 (PMC8864311; doi:10.3389/fgene.2022.762124)
Supplement: Supplementary file 17 [file Table5.DOCX]

2019-9-3-周期*6
