# Supplementary material for: Discovery of Differentially Expressed MicroRNAs in Porcine Ovaries With Smaller and Larger Litter Size
Source: Front Genet. 2022 Feb 9;13:762124. doi: 10.3389/fgene.2022.762124 (PMC8864311; doi:10.3389/fgene.2022.762124)
Supplement: Supplementary file 19 [file Table3.DOCX]

**Supplementary Material**

**Discovery of Differentially Expressed MicroRNAs in Porcine Ovaries With Smaller and Bigger Litter Size**

**Gaoxiao Xu^1,2^, Fuquan Yin^3^, Yamei Hu^2^,** **Xiao Li^2^, Saixing Duan^1^, Ning Zhang^4^, Gaoyu Xu^1^, Jianhong Hu^2^, Gongshe Yang^2^, Shiduo Sun^2^ and Xingfa Chen^2,4,*^**

**Running title: Differentially Expressed MicroRNAs in Pig Ovary**

^1^*Teaching and Research Section of Biotechnology, Nanning University, Nanning 530200, China.*

^2^*Shaanxi Key Laboratory of Molecular Biology for Agriculture, College of Animal Science and Technology, Northwest A&F University, Shaanxi 712100, China.*

^3^*College of Coastal Agricultural Science, Guangdong Ocean University, Zhanjiang* *524005, China.*

^4^*Nanning Dabeinong Feed Technology Co., Ltd., Nanning530105, China.*

**Correspondence to: Xingfa Chen (chenxf19821215@126.com) and Shiduo Sun (ssdsm@tom.com).*

**Table S1** MiRNAs identified by transcriptome sequencing.

**Table S2** Up-regulated and down-regulated of miRNAs.

**Table S1** MiRNAs identified by transcriptome sequencing.

| **AccID** | **log2FC** | **FoldChange** | **FDR** | **Normalized_gcz** | **Normalized_dcz** | **Style** | **LLS1** | **LLS2** | **LLS3** | **SLS1** | **SLS2** | **SLS3** |
| --- | --- | --- | --- | --- | --- | --- | --- | --- | --- | --- | --- | --- |
| ssc-miR-421-3p | 0.76007 | 1.693573 | 4.11E-15 | 311.396 | 183.8652 | up | 280 | 312 | 328 | 279 | 111 | 207 |
| ssc-miR-141 | -1.35376 | 0.391271 | 8.28E-07 | 436.4802 | 1115.562 | down | 527 | 321 | 425 | 1774 | 629 | 1259 |
| ssc-miR-450b-5p | 0.666935 | 1.587697 | 5.91E-09 | 7991.538 | 5033.412 | up | 8132 | 7388 | 7975 | 6600 | 3733 | 5411 |
| ssc-miR-200b | -1.41555 | 0.374868 | 7.44E-06 | 862.5365 | 2300.924 | down | 1069 | 567 | 876 | 3717 | 1272 | 2588 |
| ssc-miR-429 | -1.23771 | 0.424044 | 1.17E-10 | 36.02794 | 84.97625 | down | 44 | 23 | 38 | 119 | 54 | 100 |
| ssc-miR-1343 | 0.834688 | 1.783471 | 0.020912 | 580.7393 | 325.6186 | up | 659 | 480 | 560 | 486 | 214 | 344 |
| ssc-miR-2366 | 1.16894 | 2.248464 | 0 | 254.5794 | 113.2181 | up | 247 | 258 | 245 | 133 | 96 | 115 |
| ssc-miR-143-5p | -0.91568 | 0.530094 | 0.011686 | 1761.168 | 3322.377 | down | 2112 | 1256 | 1770 | 5284 | 1917 | 3674 |
| ssc-miR-185 | 0.773882 | 1.709864 | 4.6E-05 | 4644.01 | 2716.007 | up | 4926 | 3978 | 4724 | 3551 | 2033 | 2897 |
| ssc-miR-1 | -0.88201 | 0.542612 | 0.001085 | 6748.538 | 12437.15 | down | 8426 | 4510 | 6711 | 17489 | 8575 | 13419 |
| ssc-miR-129b | 1.90022 | 3.7327 | 0 | 2775.545 | 743.5683 | up | 2177 | 3187 | 2876 | 705 | 718 | 757 |
| ssc-miR-135 | -1.2762 | 0.41288 | 5E-10 | 813.7693 | 1970.971 | down | 969 | 603 | 803 | 2977 | 1228 | 2166 |
| ssc-miR-96-5p | 1.163191 | 2.239522 | 0.002337 | 1253.545 | 559.7323 | up | 842 | 1586 | 1311 | 484 | 583 | 539 |
| ssc-miR-497 | -0.86695 | 0.548304 | 1.18E-13 | 3036.889 | 5538.702 | down | 3101 | 2780 | 3046 | 7961 | 3724 | 5983 |
| ssc-miR-100 | -0.98529 | 0.505124 | 0.039504 | 168.345 | 333.2846 | down | 155 | 183 | 159 | 579 | 171 | 361 |
| ssc-miR-214-5p | -0.72414 | 0.605358 | 2.82E-08 | 4287.971 | 7083.371 | down | 4511 | 3751 | 4326 | 10124 | 4751 | 7723 |
| ssc-miR-149 | -0.67901 | 0.624594 | 0.00273 | 3784.194 | 6058.653 | down | 3458 | 3882 | 3834 | 9485 | 3575 | 6700 |
| ssc-miR-210 | -0.97116 | 0.510095 | 0.000259 | 1419.161 | 2782.162 | down | 1406 | 1345 | 1426 | 4481 | 1540 | 3138 |
| ssc-miR-145-3p | -1.00478 | 0.498347 | 0.017073 | 5008.313 | 10049.87 | down | 5436 | 4215 | 5031 | 16797 | 5258 | 11308 |
| ssc-miR-503 | 1.350453 | 2.549921 | 0 | 2361.389 | 926.0576 | up | 2173 | 2438 | 2360 | 1000 | 837 | 931 |
| ssc-miR-362 | 0.592225 | 1.50757 | 0 | 1309.675 | 868.729 | up | 1246 | 1266 | 1349 | 1088 | 667 | 941 |
| ssc-miR-19a | -0.66375 | 0.631234 | 0 | 1152.548 | 1825.872 | down | 1023 | 1215 | 1169 | 2475 | 1304 | 1976 |
| ssc-miR-194a-5p | -1.23027 | 0.426238 | 0 | 106.2477 | 249.2817 | down | 119 | 83 | 109 | 352 | 167 | 276 |
| ssc-miR-143-3p | -0.65296 | 0.635976 | 1.46E-05 | 354347 | 557170.4 | down | 357281 | 326991 | 357892 | 705593 | 431613 | 589941 |
| ssc-miR-182 | 1.353711 | 2.555686 | 0 | 4025.906 | 1575.268 | up | 3710 | 4124 | 4050 | 1790 | 1406 | 1534 |
| ssc-miR-192 | -0.93418 | 0.523341 | 0.00065 | 274.5809 | 524.6785 | down | 249 | 279 | 283 | 601 | 450 | 538 |
| ssc-miR-7857-3p | 3.050057 | 8.282446 | 0 | 962.0565 | 116.1473 | up | 975 | 910 | 944 | 88 | 128 | 111 |
| ssc-miR-1249 | 1.606029 | 3.044128 | 0.001415 | 518.3626 | 170.2761 | up | 632 | 364 | 515 | 277 | 101 | 178 |
| ssc-miR-129a-5p | 1.871278 | 3.658566 | 0 | 2854.111 | 780.1102 | up | 2261 | 3366 | 2844 | 755 | 750 | 786 |
| ssc-miR-99a-5p | -0.98297 | 0.505938 | 0.030544 | 25034.26 | 49480.92 | down | 20396 | 28130 | 25702 | 83095 | 26461 | 54337 |
| ssc-miR-183 | 1.533823 | 2.89552 | 9.99E-13 | 1512.476 | 522.3437 | up | 1132 | 1805 | 1560 | 507 | 494 | 539 |
| ssc-miR-10391 | 1.864491 | 3.641393 | 1.78E-05 | 1412.897 | 388.0027 | up | 1597 | 1105 | 1432 | 33 | 585 | 336 |
| ssc-miR-1842 | 0.720056 | 1.647246 | 0.019624 | 235.7201 | 143.0956 | up | 224 | 239 | 232 | 119 | 145 | 149 |

**Table S2** Up-regulated and down-regulated of miRNAs.

| **AccID** | **log2FC** | **FoldChange** | **FDR** | **Normalized_gcz** | **Normalized_dcz** | **Style** | **ZD5** | **ZD7** | **ZD8** | **ZD12** | **ZD2** | **ZD3** |
| --- | --- | --- | --- | --- | --- | --- | --- | --- | --- | --- | --- | --- |
| ssc-miR-421-3p | 0.760070042 | 1.693572845 | 4.10783E-15 | 311.3960479 | 183.8652013 | up | 280 | 312 | 328 | 279 | 111 | 207 |
| ssc-miR-450b-5p | 0.666935232 | 1.587696583 | 5.9133E-09 | 7991.537654 | 5033.41247 | up | 8132 | 7388 | 7975 | 6600 | 3733 | 5411 |
| ssc-miR-1343 | 0.834688094 | 1.783471416 | 0.020911896 | 580.7392581 | 325.6185763 | up | 659 | 480 | 560 | 486 | 214 | 344 |
| ssc-miR-2366 | 1.168940043 | 2.248464402 | 0 | 254.5793609 | 113.2181039 | up | 247 | 258 | 245 | 133 | 96 | 115 |
| ssc-miR-185 | 0.773881874 | 1.709864349 | 4.60227E-05 | 4644.010382 | 2716.006849 | up | 4926 | 3978 | 4724 | 3551 | 2033 | 2897 |
| ssc-miR-129b | 1.900219665 | 3.732700264 | 0 | 2775.544801 | 743.5682691 | up | 2177 | 3187 | 2876 | 705 | 718 | 757 |
| ssc-miR-96-5p | 1.163190928 | 2.239522139 | 0.002336754 | 1253.545281 | 559.7323036 | up | 842 | 1586 | 1311 | 484 | 583 | 539 |
| ssc-miR-503 | 1.350452563 | 2.549921021 | 0 | 2361.38923 | 926.057596 | up | 2173 | 2438 | 2360 | 1000 | 837 | 931 |
| ssc-miR-362 | 0.592225276 | 1.507570297 | 0 | 1309.675061 | 868.7289658 | up | 1246 | 1266 | 1349 | 1088 | 667 | 941 |
| ssc-miR-182 | 1.353710508 | 2.55568585 | 0 | 4025.90622 | 1575.268206 | up | 3710 | 4124 | 4050 | 1790 | 1406 | 1534 |
| ssc-miR-7857-3p | 3.050056839 | 8.282445693 | 0 | 962.0564729 | 116.1472932 | up | 975 | 910 | 944 | 88 | 128 | 111 |
| ssc-miR-1249 | 1.606029195 | 3.044128363 | 0.001414616 | 518.3625987 | 170.2760513 | up | 632 | 364 | 515 | 277 | 101 | 178 |
| ssc-miR-129a-5p | 1.871278286 | 3.658566001 | 0 | 2854.111058 | 780.1101501 | up | 2261 | 3366 | 2844 | 755 | 750 | 786 |
| ssc-miR-183 | 1.533822532 | 2.895520141 | 9.98979E-13 | 1512.475556 | 522.3436643 | up | 1132 | 1805 | 1560 | 507 | 494 | 539 |
| ssc-miR-10391 | 1.864490575 | 3.641393307 | 1.77942E-05 | 1412.896851 | 388.0027007 | up | 1597 | 1105 | 1432 | 33 | 585 | 336 |
| ssc-miR-1842 | 0.720055906 | 1.647245866 | 0.01962407 | 235.7200993 | 143.0955947 | up | 224 | 239 | 232 | 119 | 145 | 149 |
| ssc-miR-141 | -1.35376174 | 0.391270504 | 8.27587E-07 | 436.4802365 | 1115.561536 | down | 527 | 321 | 425 | 1774 | 629 | 1259 |
| ssc-miR-200b | -1.415545594 | 0.374867954 | 7.44279E-06 | 862.5364559 | 2300.924093 | down | 1069 | 567 | 876 | 3717 | 1272 | 2588 |
| ssc-miR-429 | -1.23771289 | 0.424044363 | 1.16939E-10 | 36.02793997 | 84.97624942 | down | 44 | 23 | 38 | 119 | 54 | 100 |
| ssc-miR-143-5p | -0.915679034 | 0.530094312 | 0.011685533 | 1761.168331 | 3322.376772 | down | 2112 | 1256 | 1770 | 5284 | 1917 | 3674 |
| ssc-miR-1 | -0.882007784 | 0.542611758 | 0.00108521 | 6748.537854 | 12437.14742 | down | 8426 | 4510 | 6711 | 17489 | 8575 | 13419 |
| ssc-miR-135 | -1.276204557 | 0.412880287 | 4.99748E-10 | 813.7693466 | 1970.971353 | down | 969 | 603 | 803 | 2977 | 1228 | 2166 |
| ssc-miR-497 | -0.866951774 | 0.548304123 | 1.18239E-13 | 3036.888518 | 5538.701807 | down | 3101 | 2780 | 3046 | 7961 | 3724 | 5983 |
| ssc-miR-100 | -0.985291815 | 0.505123541 | 0.039503635 | 168.3449548 | 333.2846122 | down | 155 | 183 | 159 | 579 | 171 | 361 |
| ssc-miR-214-5p | -0.724139649 | 0.605357942 | 2.8228E-08 | 4287.971059 | 7083.371187 | down | 4511 | 3751 | 4326 | 10124 | 4751 | 7723 |
| ssc-miR-149 | -0.679009767 | 0.624593834 | 0.002730236 | 3784.193527 | 6058.652959 | down | 3458 | 3882 | 3834 | 9485 | 3575 | 6700 |
| ssc-miR-210 | -0.971163307 | 0.510094586 | 0.000258749 | 1419.160691 | 2782.161642 | down | 1406 | 1345 | 1426 | 4481 | 1540 | 3138 |
| ssc-miR-145-3p | -1.004778449 | 0.498346656 | 0.017073051 | 5008.312633 | 10049.86708 | down | 5436 | 4215 | 5031 | 16797 | 5258 | 11308 |
| ssc-miR-19a | -0.663754233 | 0.631233539 | 0 | 1152.547803 | 1825.871756 | down | 1023 | 1215 | 1169 | 2475 | 1304 | 1976 |
| ssc-miR-194a-5p | -1.230267418 | 0.426238431 | 0 | 106.2476914 | 249.2816726 | down | 119 | 83 | 109 | 352 | 167 | 276 |
| ssc-miR-143-3p | -0.652955822 | 0.635975978 | 1.46453E-05 | 354346.9924 | 557170.4095 | down | 357281 | 326991 | 357892 | 705593 | 431613 | 589941 |
| ssc-miR-192 | -0.934177267 | 0.523340833 | 0.000649622 | 274.5809317 | 524.6785288 | down | 249 | 279 | 283 | 601 | 450 | 538 |
| ssc-miR-99a-5p | -0.982968483 | 0.505937653 | 0.030543514 | 25034.25696 | 49480.9227 | down | 20396 | 28130 | 25702 | 83095 | 26461 | 54337 |
